# Application of Multiscale Sample Entropy in Assessing Effects of Exercise Training on Skin Blood Flow Oscillations in People with Spinal Cord Injury

**DOI:** 10.3390/e25040690

**Published:** 2023-04-19

**Authors:** Fuyuan Liao, Hengyang Zhao, Cheng-Feng Lin, Panpan Chen, Philbert Chen, Kingsley Onyemere, Yih-Kuen Jan

**Affiliations:** 1 Department of Biomedical Engineering, Xi’an Technological University, Xi’an 710021, China; 2 School of Electronic Information Engineering, Xi’an Technological University, Xi’an 710021, China; 3 Rehabilitation Engineering Lab, Department of Kinesiology and Community Health, University of Illinois at Urbana-Champaign, Urbana, IL 61801, USA; 4 Carle Foundation Hospital, Urbana, IL 61801, USA

**Keywords:** exercise training, microvascular function, multiscale entropy, regularity, skin blood flow

## Abstract

Spinal cord injury (SCI) causes a disruption of autonomic nervous regulation to the cardiovascular system, leading to various cardiovascular and microvascular diseases. Exercise training is an effective intervention for reducing risk for microvascular diseases in healthy people. However, the effectiveness of exercise training on improving microvascular function in people with SCI is largely unknown. The purpose of this study was to compare blood flow oscillations in people with spinal cord injury and different physical activity levels to determine if such a lifestyle might influence skin blood flow. A total of 37 participants were recruited for this study, including 12 athletes with SCI (ASCI), 9 participants with SCI and a sedentary lifestyle (SSCI), and 16 healthy able-bodied controls (AB). Sacral skin blood flow (SBF) in response to local heating at 42 °C for 50 min was measured using laser Doppler flowmetry. The degree of the regularity of blood flow oscillations (BFOs) was quantified using a multiscale entropy approach. The results showed that BFO was significantly more irregular in ASCI and AB compared to SSCI during the maximal vasodilation period. Our results also demonstrate that the difference in the regularity of BFOs between original SBF signal and phase-randomized surrogate time series was larger in ASCI and AB compared to SSCI. Our findings indicate that SCI causes a loss of complexity of BFOs and exercise training may improve complexity in people with SCI. This study demonstrates that multiscale entropy is a sensitive method for detecting differences between different categories of people with SCI and might be able to detect effects of exercise training related to skin blood flow.

## 1. Introduction

Spinal cord injury (SCI) causes a disruption of autonomic nervous regulation to the cardiovascular system, leading to various cardiovascular and microvascular diseases [1]. People with SCI usually have fewer opportunities to participate in physical activities and exercise training, which results in increased intramuscular fat for a higher risk for metabolic diseases [2]. These cardiovascular and metabolic diseases further aggravate microvascular function in people with SCI, which results in impaired microvascular response to thermal, mechanical and exercise stresses [3,4,5].

Exercise training is an effective intervention for reducing risks for cardiovascular and metabolic diseases in healthy people [6]. However, the effectiveness of exercise training (regular daily exercise) on improving cardiovascular function in people with SCI is largely unclear due to limited evidence in the literature [7]. Due to muscle paralysis of the lower limb, people with SCI usually use a wheelchair as a primary means of mobility and can only perform upper-limb exercise training. However, it is unclear whether upper-body exercise training can effectively improve cardiovascular function in people with SCI [8]. Totosy de Zepetnek et al. [9] demonstrated that 16 weeks of upper-body exercise training did not significantly change blood vessel stiffness and flow-mediated dilation of the carotid artery in people with SCI. Jansen et al. [10] demonstrated that there were no changes in the intima–media thickness of the common coronary artery and superficial femoral artery and flow-mediated dilation of the brachial artery after 16 weeks of hybrid cycling training (i.e., leg cycling with electric stimulation combined with voluntary hand cycling, or hand cycling) in people with SCI. These studies focused on the influence of exercise training on conduit artery function and did not assess microvascular function that is an essential component in regulating body temperature under thermal stresses and tissue viability under ischemic stresses. Furthermore, cardiovascular function is regulated by the autonomic nervous system for maintaining a sympathovagal balance on modulating heart rate to meet the demands of our body [11,12]. The study of heart rate variability after spinal anesthesia and spinal cord injury has been used to investigate the degree of sympathetic and parasympathetic dysfunction in people with SCI as well as to assess the efficacy of education training [11,13,14]. To sum up, the study of cardiovascular function, including microvascular function, would allow clinicians to understand the influence of SCI and exercise training on improving health in the SCI population.

Skin blood flow (SBF) function is a promising assessment to quantify the benefits of exercise training in people with SCI. SBF has been assessed using laser Doppler flowmetry (LDF), a noninvasive optical device. LDF SBF signals are usually quantified using the time domain, time–frequency domain, and nonlinear indices [15,16]. Wavelet analysis, a common time–frequency domain approach, of laser Doppler SBF signals reveals six frequency components [17]. Two components at higher frequencies are related to cardiac (0.4–2 Hz) and respiratory (0.15–0.4 Hz) activities and the other components are associated with myogenic (0.05–0.15 Hz), neurogenic (0.02–0.05 Hz), and endothelial (0.0095–0.02 Hz and 0.005–0.0095 Hz) activities of the vessel wall [17,18]. The mean amplitude or power of each frequency component has been used to characterize the state of the regulatory mechanisms of SBF [15,16]. In our previous study [18], we examined sacral SBF responses after thermal stresses in wheelchair users with SCI and found that a smaller response of SBF to externally applied pressure with local cooling compared to pressure alone and pressure with local heating. By employing wavelet analysis, we found that the smaller SBF response induced by pressure with local cooling was due to attenuated metabolic and neurogenic activities [3,4,18].

Although time domain indices provide a direct depiction of SBF response and wavelet analysis provides a straightforward interpretation of the underlying mechanisms, they are incapable of revealing nonlinear features, e.g., self-similarity and complexity, of blood flow oscillations (BFOs) [5,16,19]. Complexity analysis has been used to characterize cardiovascular control mechanisms [19]. Porta et al. demonstrated that complexity analysis using a local version of sample entropy is more sensitive to detect the decrease in complexity of heart rate variability. The use of complexity analysis, such as sample entropy, could quantify the change in the complexity of BFOs following SCI. In our previous studies [20,21], we observed a higher degree of the irregularity of BFOs in the plantar foot of diabetics compared to healthy controls during reactive hyperemia, whereas the relative amplitudes of all frequency components did not show significant differences between two groups. This discrepancy may be due to the fact that the degree of the regularity of BFOs could reflect the interactions among the regulatory mechanisms of SBF, whereas relative amplitudes of the frequency components characterize the activity levels of the mechanisms. Therefore, there is a need to assess time domain, time–frequency domain and nonlinear domain of microvascular function in people with SCI.

To date, most studies use the time domain and time–frequency domain approaches to assess BFOs in people with SCI, and only a few studies use a nonlinear approach to assess BFOs in people with SCI [20,21,22]. There is no study assessing the effect of exercise training on BFOs in people with SCI using a nonlinear approach. Therefore, the objective of this study was to compare blood flow oscillations in people with spinal cord injury and different physical activity levels to determine if such a lifestyle might influence skin blood flow using a multiscale sample entropy approach. Because our previous studies have validated the feasibility of using multiscale entropy to quantify regularity degree of BFOs [20,21,22], we intended to utilize this method to investigate whether exercise training could improve complexity of BFOs in people with SCI.

## 2. Methods

This study used a pre–post study design in three participant groups. The intervention is a fast local heating protocol to induce a maximal vasodilatory response. The participant groups included people with SCI (physically active and sedentary lifestyle) and non-SCI controls. The outcome measure was sacral skin blood flow, the area at high risk for pressure injury and chronic wounds. The multiscale sample entropy was used to quantify the regularity of BFOs before heating and under heating in three participant groups. This human subject research was approved by the Institutional Review Board (OUHSC #14313).

### 2.1. Subjects

The inclusion criteria for participants with SCI included the injury level between C4 and T12, spinal injury occurred more than 6 months, age between 18 and 50 years, and not taking medications that might affect cardiovascular function. The ASCI group was limited to people with SCI who participated in exercise training at least 4 times a week for more than 1 year prior to participating this study. The SSCI group was limited to people with SCI who did not participate in any physical activity and exercise training for more than 1 year prior to participating in this study. All participants gave their informed consent. The criteria used in this study were to recruit distinct cases (either very physically active or very sedentary life style) from the SCI population. This study recruited 37 participants, including 12 athletes with SCI (ASCI group), 9 participants with SCI who lived a sedentary lifestyle (SSCI group), and 16 able-bodied controls (AB group). Their demographic data are shown in Table 1.

### 2.2. Experimental Protocol

All experiments were performed in a university research laboratory. Room temperature was maintained at 24 ± 2 °C. Prior to the experiment, the participant stayed in the laboratory for at least 30 min to acclimate to the room temperature. Then, the subject lied in a prone posture. Sacral skin blood flow was measured using a laser Doppler flowmetry (PeriFlux 5001, Perimed, Las Vegas, NV, USA) with a probe with heating function (Probe 415–242, Perimed). The sampling rate was 32 Hz. The experimental protocol consisted of a 10 min baseline measurement, followed by a 50-min heating period at 42 °C, and a 10-min recovery period [23].

### 2.3. Time Domain Assessment of SBF

The local heating procedure induced a biphasic vasodilatory response, during which SBF initially rises rapidly followed by a nadir and then increases slowly to a plateau [23]. This response has been quantified by the biphasic thermal index, including three ratios, i.e., ratios of the first peak, nadir, and second peak to baseline blood flow [24]. Since SBF rises to the maximal level and exhibits little change during the latter epoch of heating, the last 10-min epoch is considered as the second peak (i.e., maximal vasodilatory response) [23].

### 2.4. Multiscale Sample Entropy Analysis

Sample entropy ($E_{s}$) quantifies the degree of the regularity/irregularity of a sequence by measuring the conditional probability that two different subsequences of m points within a threshold r remain within the threshold at the next point [25,26]. A smaller value of $E_{s}$ indicates a lower degree of irregularity and vice versa. An outstanding advantage of $E_{s}$ over other nonlinear measures is its robustness when applied to short and noisy data series [25,26]. However, as has been demonstrated in previous studies [22,25,26], for a given data series, $E_{s}$ depends not only on the regularity degree itself but also on the relationship between the dominated frequency and the sampling rate. If the latter is much higher than the former, $E_{s}$ may not be able to reflect the actual regularity degree of the data series. To address this problem, we used a modified sample entropy ($E_{ms}$) algorithm [22,25,26]. Its procedures are described briefly as below.

For a sequence of length N, {x(i),i=1,…,N}, consider m-point vectors $x_{m}^{\tau }(i)=\{x(i+k\tau ),0\leq k\leq m-1\}$, 1≤i≤N−mτ, where τ is a delay in data points, determined as the first minimum of the mutual information (MI) function of the sequence [22,27]. The distance between two vectors, $x_{m}^{\tau }(i)$ and $x_{m}^{\tau }(j)$, is defined as

(1) $$d[x_{m}^{\tau }(i),x_{m}^{\tau }(j)]=\max\{|x(i+k\tau )-x(j+k\tau )|,0\leq k\leq m-1\}$$

For a vector $x_{m}^{\tau }(i)$, let $n_{i}^{m}(r)$ be the number of vectors $x_{m}^{\tau }(j)$ satisfying the condition

(2) $$d[x_{m}^{\tau }(i),x_{m}^{\tau }(j)]<r,\;|j-i|>\tau$$

**Figure 1:**
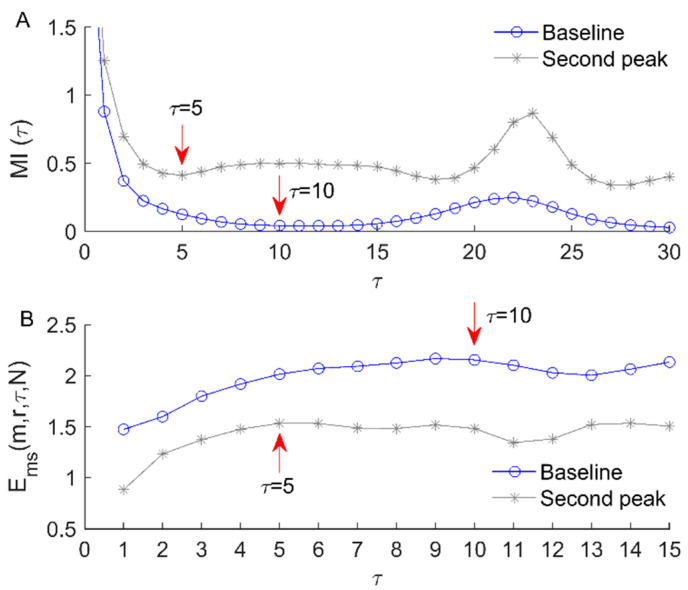
Illustration of the relationship between delay τ and entropy $E_{ms}(m,r,\tau ,N)$ for SBF signals. (**A**) Examples of mutual information function MI(τ) of a SBF signal during the baseline and second peak periods. The values of τ determined as the first minima of MI(τ) for the two signal epochs are 10 and 5, respectively. (**B**) $E_{ms}(m,r,\tau ,N)$ increases initially with increasing values of τ and when τ takes a value around that determined as the first minimum of MI(τ).

Hence, $C^{m}(r)=\frac{1}{N-m\tau }\sum_{i=1}^{N-m\tau }n_{i}^{m}(r)$ represents the probability that any two vectors, $x_{m}^{\tau }(i)$ and $x_{m}^{\tau }(j)$, |j−i|>τ, are within the threshold r. Likewise, let $C^{m+1}(r)$ represent the probability that any two vectors, $x_{m+1}^{\tau }(i)$ and $x_{m+1}^{\tau }(j)$, |j−i|>τ, are within the threshold r. The modified sample entropy of the sequence {x(i),i=1,…,N} is estimated by the statistic

(3) $$E_{ms}(m,r,\tau ,N)=-\ln\frac{C^{m+1}(r)}{C^{m}(r)}$$

where r is set be proportional to the standard deviation (SD) of the sequence. Obviously, $E_{ms}$ is a multiscale entropy measure when τ takes multiple values and degenerates to $E_{s}$ when τ = 1. Typically, $E_{ms}$ initially increases with increasing τ and achieves a plateau when τ takes the value at which MI(τ) reaches its first minimum. Figure 1 shows two examples. Thus, we consider the τ value determined by the first minimum of MI(τ) as the optimal value [22,27].

**Figure 2:**
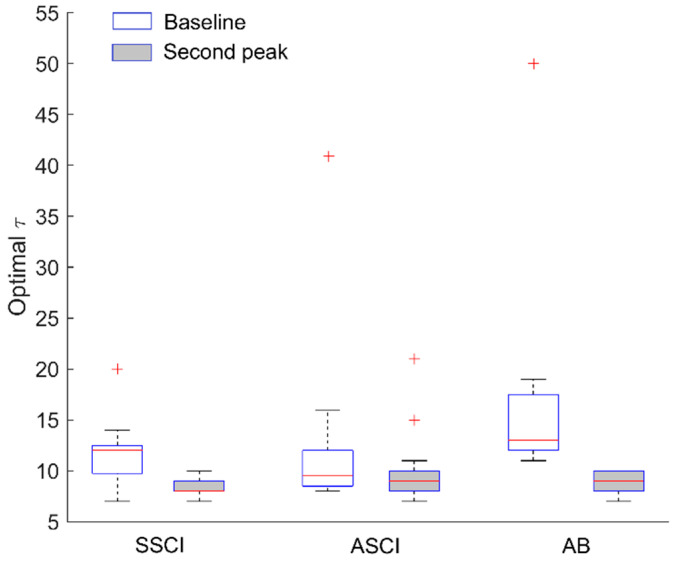
The optimal values of τ, determined as the first minimum of MI(τ), in three groups for SBF signals during the baseline and second peak periods.

The performance of $E_{ms}$ has been systematically tested using simulated time series and SBF data [21,22]. The testing results showed that $E_{ms}$ is able to reflect changes in structural properties of SBF oscillations and shows relative consistency. Moreover, if time delay τ in data points takes the value determined by the first minimum of MI(τ), $E_{ms}$ is almost independent of the sampling rate [22,25,26]. We computed $E_{ms}(m,r,\tau ,N)$ for each SBF signal epoch. The parameters m = 2 and r = 0.2 × SD, where SD is the standard deviation of the signal epoch, were used according to previous studies [20,21,22,28,29]. As for the parameter τ, we first computed its optimal value for each signal epoch and then selected an upper limit according to the distribution of the optimal τ values for all signal epochs. As shown in Figure 2, for the SBF signals during the baseline period, the medians of the optimal values of τ in three groups ranging from 10 to 13, while for the signals during the second period, the median of the optimal values is approximately 7. Therefore, we selected 15 as the upper limit of the τ values. The same value of τ was used when comparing $E_{ms}$ among three groups.

### 2.5. Surrogate Tests

In order to explore insight into the nonlinearity of SBF oscillations in people with SCI, we performed surrogate tests for the SBF signals. For each SBF signal epoch, 30 surrogate time series were generated using the iterated amplitude adjusted Fourier transform algorithm [30], in which the power spectrum of the original signal was preserved but the nonlinear structures were destroyed [30]. An index that indicates the nonlinearity of the signal epoch is defined as [31].

(4) $$\sigma =\frac{av[E_{ms}^{\left(s\right)}]-E_{ms}^{\left(o\right)}}{2\cdot sd[E_{ms}^{\left(s\right)}]}$$

where $E_{ms}^{\left(s\right)}$ and $E_{ms}^{\left(o\right)}$ represent $E_{ms}$ of surrogate time series and that of the original signal epoch, respectively; $av[E_{ms}^{\left(s\right)}]$ and $sd[E_{ms}^{\left(s\right)}]$ represent the average and standard deviation of $E_{ms}^{\left(s\right)}$ values, respectively.

### 2.6. Statistical Analysis

The normality of the distributions of all indices was tested using the Shapiro–Wilk test. Since each index obeys normal distribution, the differences in all indices between the baseline and second peak periods were examined using the paired *t* test; the differences in all indices between three groups were examined using one-way ANOVA and the *t* test. Bonferroni corrections were used for multiple comparisons. The significance level was set at 0.05.

## 3. Results

**Figure 3:**
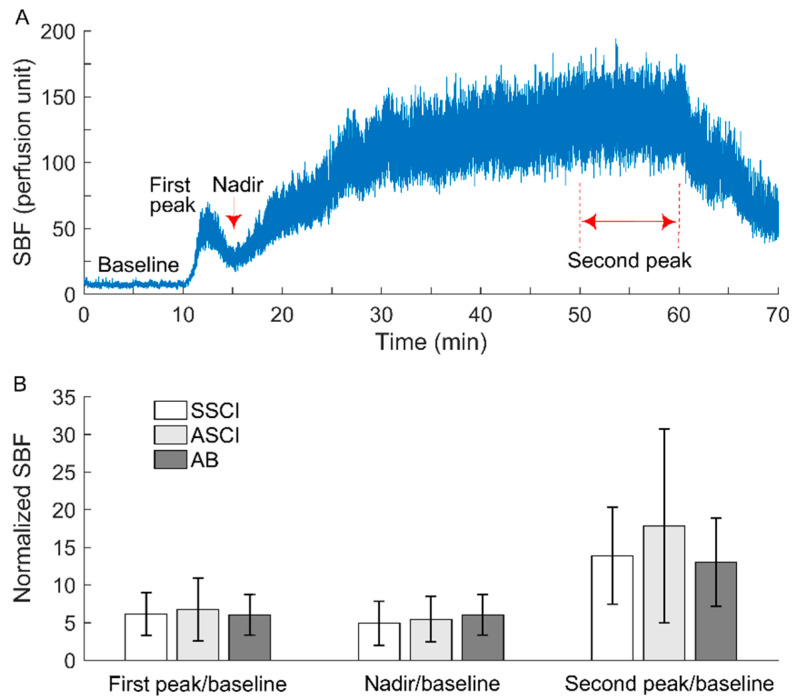
(**A**) A typical SBF response to local heating. (**B**) Biphasic thermal index (ratios of the first peak, nadir, and second peak to baseline) in three groups. The results are presented as the mean ± standard deviation. The differences in three ratios between three groups were examined using one-way ANOVA and the *t* test but no significant difference was observed.

A typical SBF response is shown in Figure 3A. Figure 3B shows the statistical results of biphasic thermal index. No significant difference was observed.

**Figure 4:**
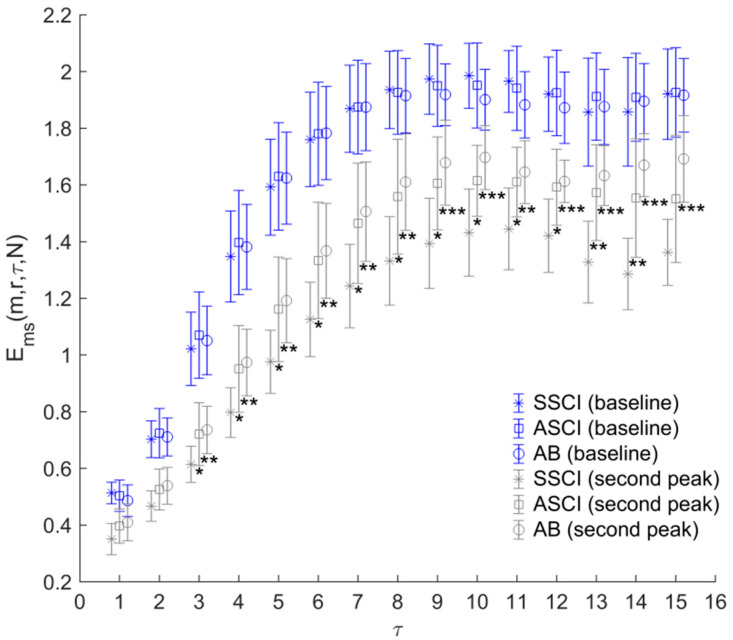
Comparisons of $E_{ms}(m,r,\tau ,N)$ between the baseline and second peak periods and between three groups. The results are presented as the mean ± standard deviation. For the within-group comparisons, $E_{ms}(m,r,\tau ,N)$ in all groups significantly reduced during the second peak period for τ from 1 to 15 (*p* < 0.0001) in the SSCI and AB groups and *p* < 0.01 in the ASCI group (paired *t*-test). For the between-group comparisons, only during the second peak period, $E_{ms}(m,r,\tau ,N)$ was significantly larger in the AB and ASCI groups than in the SSCI group for most values of τ (ANOVA and *t*-test). *, *p* < 0.05; **, *p* < 0.01; ***, *p* < 0.001.

Figure 4 shows the statistical results of $E_{ms}$ of the SBF signals during the baseline and second peak periods in three groups. In all groups, $E_{ms}$ significantly reduced during the second peak period compared to the baseline period for τ from 1 to 15. For the between-group comparisons, only during the second peak period, significant differences in $E_{ms}$ were observed. Compared to the SSCI group, $E_{ms}$ yielded significantly larger values in the AB and ASCI groups for τ from 3 to 15. No significant difference in $E_{ms}$ was observed between the ASCI and AB groups.

**Figure 5:**
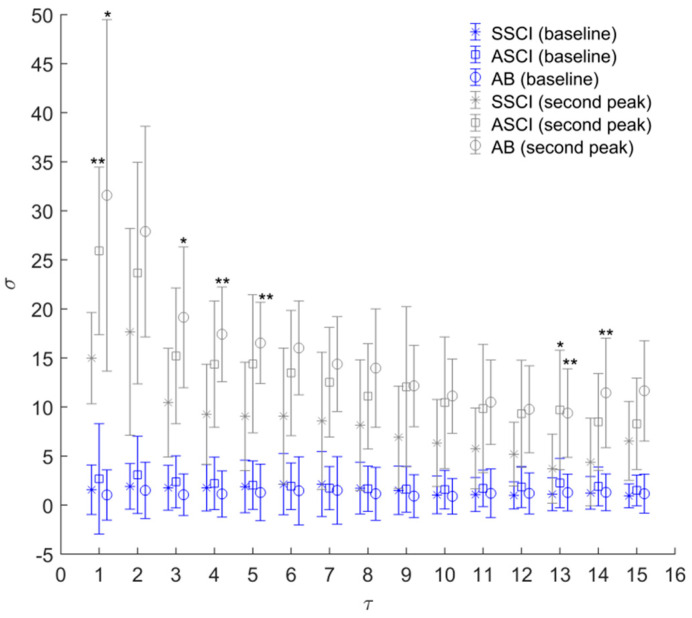
Statistical results of σ (Equation (4)). The results are presented as the mean ± standard deviation. * and **, respectively, indicate *p* < 0.05 and *p* < 0.01 for comparisons between the SSCI and ASCI groups and between the SSCI and AB groups.

Figure 5 shows the statistical results of σ (Equation (4)). In all groups, σ was significantly larger during the second peak compared to the baseline. For the between-group comparisons, only during the second peak period, significant differences in σ were observed. Compared to SSCI group, σ in the ASCI and AB groups was significantly larger for several values of τ.

## 4. Discussion

This study demonstrates that BFOs in all groups were significantly more regular during the second peak period of SBF response induced by local heating compared to the baseline period. The degree of regularity in the ASCI group was similar to that in the AB group and significantly lower than in the SSCI group. Second, during the second peak period, the difference in regularity degree between the original SBF signal and surrogate time series in the ASCI group was also similar to that in the AB group and larger than in the SSCI group. These findings implied that during the second peak period of the SBF response, the interactions among the regulatory mechanisms of SBF were attenuated in SSCI subjects but improved in ASCI subjects, suggesting that physical activity and exercise training could improve skin microvascular function in people with SCI.

In this study, we employed a modified sample entropy ($E_{ms}(m,r,\tau ,N)$) method [22,25,26] to quantify the regularity degree of skin BFOs. Our previous studies [21,22] have demonstrated that $E_{ms}$ is actually a multiscale entropy measure for varying values of τ [21]. As shown in Figure 4, a common feature of $E_{ms}$ of SBF signals was that $E_{ms}$ initially increased with increasing scale and then reached a plateau, indicating that for a certain range of scales, BFOs exhibited more complex dynamics at larger scales. It is worth noting that only at the larger scales were there significant differences in $E_{ms}$ between three groups. This means that the traditional sample entropy, i.e., $E_{ms}$ when τ = 1, is incapable of quantifying the regularity degree of the main oscillatory components of SBF signals.

**Figure 6:**
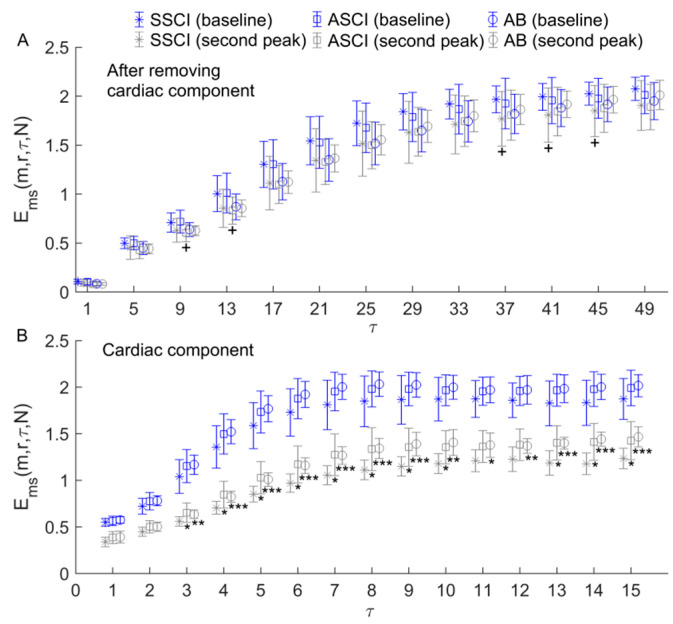
(**A**) Comparisons of $E_{ms}(m,r,\tau ,N)$ of SBF signals after removing the cardiac component between the baseline and second peak periods and between three groups. Only the results for τ = 1, 5, …, 49 are shown and presented as the mean ± standard deviation. Compared to the baseline period, $E_{ms}(m,r,\tau ,N)$ was significantly lower during the second peak period for τ from 34 to 45 in the SSCI group and for τ from 6 to 16 in the ASCI group. +, *p* < 0.05 (paired *t*-test). (**B**) Comparisons of $E_{ms}(m,r,\tau ,N)$ of the cardiac component between the baseline and second peak periods and between three groups.

Our results showed that during the second peak period of the SBF response, $E_{ms}$ at most scales between 1 and 15 was significantly larger in the ASCI group than in the SSCI group but similar to that in the AB group (Figure 4), indicating more irregular BFOs in ASCI and AB subjects compared to SSCI subjects. This observation is analogous to our previous findings that skin BFOs were more irregular in healthy young subjects than in older subjects during the maximal vasodilation period and that the more irregular behavior of BFOs was mainly due to a less enhanced cardiac component [22]. In this study, to verify whether the significant differences in $E_{ms}$ of BFOs between three groups (Figure 4) were mainly due to the differences in $E_{ms}$ of cardiac oscillations, we performed the following experiment. For each SBF signal epoch, the cardiac component was extracted by utilizing the ensemble empirical mode decomposition algorithm [32]. Then, $E_{ms}$ was computed for the cardiac component and the residual signal. The results showed that during the second peak period, cardiac oscillations in the ASCI and AB groups were significantly more irregular compared to the SSCI group (Figure 6B) and no significant difference in $E_{ms}$ of the residual signals was observed between three groups (Figure 6A). These results suggested that during the second peak period, more irregular BFOs in ASCI and AB subjects were mainly due to more irregular cardiac oscillations.

Compared to the baseline period (*t*-test), $E_{ms}(m,r,\tau ,N)$ in all groups was significantly lower during the second peak period for τ from 1 to 15 (*p* < 10^−3^ in the SSCI group, *p* < 10^−4^ in the ASCI group, and *p* < 10^−6^ in the AB group). For the between-group comparisons (ANOVA and *t*-test), during the second peak period, $E_{ms}(m,r,\tau ,N)$ in the SSCI group was significantly lower than in both the ASCI and AB groups for most τ values between 1 and 15. *, *p* < 0.05; **, *p* < 10^−2^; ***, *p* < 10^−3^.

**Figure 7:**
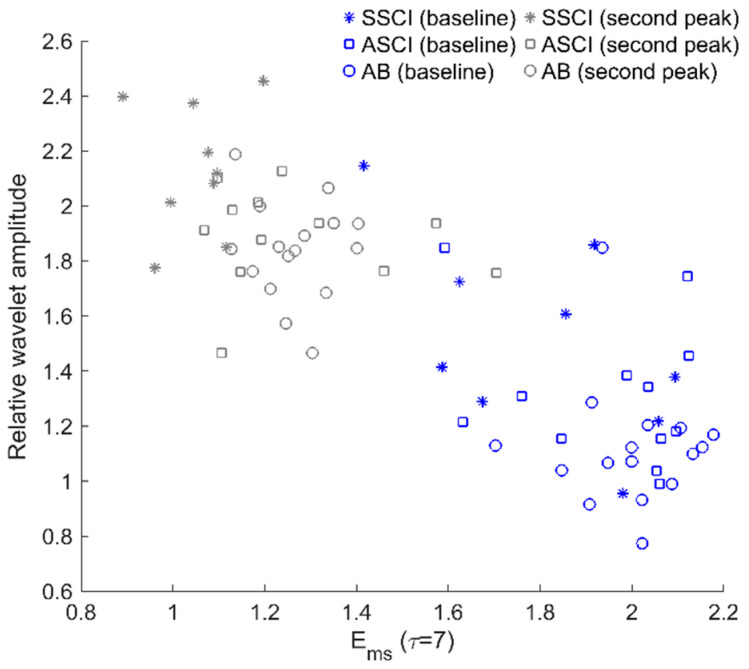
Scatterplot showing relative amplitude ($A_{r}$) and regularity degree ($E_{ms}$, τ = 7) of the cardiac component for each subject during the baseline and second peak periods.

**Figure 8:**
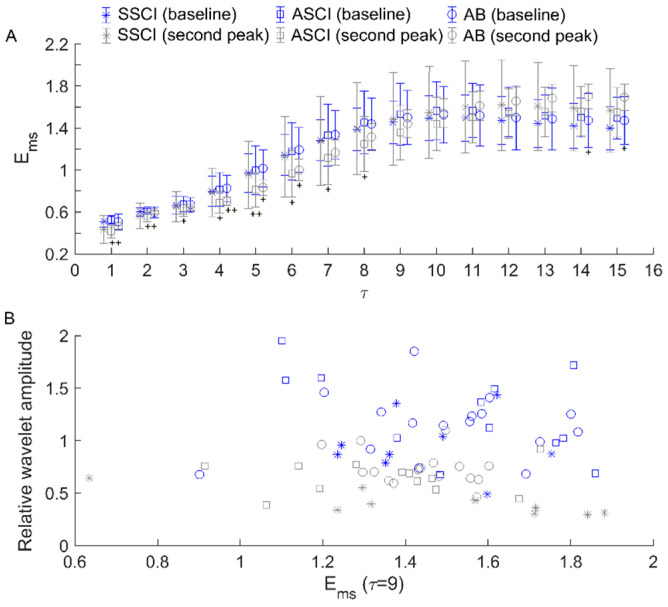
(**A**) Comparisons of $E_{ms}(m,r,\tau ,N)$ of the myogenic component (downsampled to 4 Hz) between the baseline and second peak periods and between three groups. +, *p* < 0.05; ++, *p* < 0.01 (paired *t*-test). (**B**) Scatterplot showing relative amplitude ($A_{r}$) and regularity degree ($E_{ms}$, τ = 9) of the myogenic component for each subject during the baseline and second peak periods.

To further investigate the underlying mechanisms responsible for the distinct differences in regularity degree of BFOs between three groups, we performed wavelet analysis of the SBF signals. After implementing continuous wavelet transform, we calculated the relative amplitudes ($A_{r}$) of five characteristic frequency components, i.e., metabolic, neurogenic, myogenic, respiratory, and cardiac components. This measure was defined as the ratio of averaged amplitude of a specific frequency component over time to averaged amplitude of all frequency components over time [21], aiming to quantify the relative activity levels of the regulatory mechanisms of SBF. The results showed that in all groups, $A_{r}$ of the cardiac component and $A_{r}$ of the myogenic component significantly increased and reduced, respectively, from the baseline period to second peak period. During the second peak period, $A_{r}$ of the cardiac component in the ASCI group was similar to that in the AB group and significantly lower than in the SSCI group, while $A_{r}$ of the myogenic component in the ASCI group was slightly lower than in the AB group but significantly higher than in the SSCI group. The above results indicated that during the second peak period, the cardiac component in ASCI and AB subjects differed distinctly from that in SSCI subjects not only in $E_{ms}$ but also in $A_{r}$. However, we argue that there is no association between $E_{ms}$ and $A_{r}$ because they quantify the structural and magnitude features of BFOs, respectively. As shown in Figure 7, during the second peak period, values of $E_{ms}$ of cardiac oscillations in SSCI and AB subjects were almost linearly separable, i.e., only the largest value for SSCI subjects was larger than the smallest value for AB subjects, whereas $A_{r}$ values for the two groups were linearly inseparable although the difference between two groups reached a significant level. Another case in point was that during the second peak period, $A_{r}$ of myogenic oscillations was significantly lower in the SSCI group than in the AB group but no significant difference existed in $E_{ms}$ between the two groups (Figure 8).

Our results also demonstrated that during the second peak period, σ (Equation (4)) was larger in the ASCI and AB groups than in the SSCI group. Since σ reflects nonlinear degree of BFOs, our results suggested that SCI leaded to a loss of the nonlinearity of BFOs. This finding provide a new index to assess the short-term and immediate effects of exercise on microvascular function in people with SCI. This may complement current research on investigating the effect of exercise training on heart rate variability as a surrogate assessment on the sympathovagal balance on cardiovascular function [12,13,19]. HRV has been found to be sensitive on detecting the effectiveness of exercise training on modulating cardiac autonomic control during training and long-term effect of exercise training [11]. With a combination of HRV and BFOs, researchers can explore the influence of exercise training on systematic cardiovascular function [33] and peripheral blood supply and risk for tissue ischemia and pressure ulcer in people with SCI, especially who have impaired mobility [5]. Jan et al. demonstrated that postural changes caused a significant change in HRV and sacral SBF in people with SCI [14]. Although the exact effect of HRV on BFOs remains largely unknown, the use of both HRV and BFOs can provide insight on the influences of various levels and completeness of SCI on cardiovascular function in people with SCI. Specifically, the effect of SCI on the central cardiovascular regulation and peripheral microvascular regulation and the interactions between central cardiovascular and peripheral microvascular on tissue viability and risk for pressure ulcers under externally applied pressure can be quantified. To sum up, a sensitive index to assess the effect of exercise on microvascular function could assist clinicians to determine the minimum intensity of exercise to improve microvascular function in people with SCI who are limited to participating in various physical activities due to their muscle paralysis and limited accessible exercise equipment.

There are limitations of this study. First, this study is a cross-sectional study. We did not follow the participants for monitoring the longitudinal changes in the regularity of BFOs after participating in physical activity and exercise training. According to their responses, common exercise training included wheelchair racing and wheelchair basketball for at least one hour, 4 times per week for more than one year. Most athletes with SCI had participated in state-level and/or national wheelchair games, such as National Veterans Wheelchair Games and Oklahoma Endeavor Games. Further studies need to validate our findings using a longitudinal study design. Second, we recruited participants with both complete and incomplete injury of SCI. It is unclear whether the completeness of SCI could affect the outcomes of physical activity and exercise training. Last, we used a multiscale sample entropy approach to quantify the degree of the regularity of BFOs. Future studies may use other nonlinear approaches to assess different nonlinear characteristics of BFOs.

## 5. Conclusions

Our results demonstrated that BFOs were significantly more irregular during the local heating-induced maximal vasodilation period in the ASCI and AB groups compared to in the SSCI group, and the irregularity was mainly due to the more irregular behavior of cardiac oscillations (0.4–2 Hz). Additionally, the difference in regularity between the original SBF signal and phase-randomized surrogate time series was larger in ASCI and AB compared to SSCI. These findings imply that SCI leads to an attenuated interaction among the regulatory mechanisms of sacral skin blood flow and a loss of the nonlinearity of BFOs. This study demonstrates that a multiscale entropy approach is a sensitive method for detecting differences between different categories of participants with SCI and might be able to detect effects of exercise and exercise training related to skin blood flow in subsequent research.

## Figures and Tables

**Table 1:** Demographic data of the research participants.

Demographics	SSCI	ASCI	AB
Number of subjects	9	12	16
Gender (M/F)	5/4	9/3	11/5
Age (year)	35.8 ± 11.0	35.1 ± 11.9	29.4 ± 6.2
Body mass index (kg/m^2^)	23.3 ± 2.5	25.8 ± 4.9	23.4 ± 2.9
Duration of injury (year)	9.7 ± 3.8	6.7 ± 5.9	/

Data are presented as the mean ± SD. SSCI: sedentary spinal cord injury; ASCI: athletic spinal cord injury; AB: able-bodied controls.

## Data Availability

Data will be made available on request.
